# Optimal Waveform Design Using Frequency-Modulated Pulse Trains for Active Sonar

**DOI:** 10.3390/s19194262

**Published:** 2019-09-30

**Authors:** Chengyu Guan, Zemin Zhou, Xinwu Zeng

**Affiliations:** College of Meteorology and Oceanology, National University of Defense Technology, Changsha 410000, China; guan_nudt@nudt.edu.cn

**Keywords:** active sonar, frequency-modulated, ambiguity function, waveform design, genetic algorithm

## Abstract

Frequency-modulated pulse trains can be applied in active sonar systems to improve the performance of conventional transmitted waveforms. Recently, two pulse trains have been widely researched as the transmitted waveforms for active sonars. The LFM-Costas pulse train was formed by modulating the linear frequency-modulated (LFM) waveform via the Costas sequence to remove the Doppler ambiguity of LFM pulses. The generalized sinusoidal frequency-modulated (GSFM) waveform, another frequency-modulated pulse train, achieved an ideal ambiguity function shape with thumbtack mainlobe. In this paper, we focus on constructing an optimization model to optimize the LFM-Costas and GSFM pulse trains with the genetic algorithm. The pulse trains can be improved on properties of both ambiguity function and correlations between sub-pulses. The optimized pulse trains are proven to have better detection performance than those of the initial pulse trains, including the lower sidelobe levels of ambiguity function, as well as lower cross-correlation property. Moreover, it is affirmed that the reverberation suppression performance of pulse trains has also been improved through the optimization model.

## 1. Introduction

Active sonar systems detect targets by transmitting specific signals and analyzing echoes from targets. Currently, most anti-submarine active sonars are pulsed active sonars. In order to detect remote targets in noise environments, high-energy pulses are transmitted to gain the required signal-noise ratio. For example, the source level of long-distance pulsed active sonars applied by the United States (U.S.) Navy can be as high as 235 dB [1]. Higher source levels promote the requirement on transducers, resulting in more difficult manufacturing processes and higher costs. In addition, with such a high source level, a cavitation may be induced close to the transducers in shallow water, which would cause corrosion and changes in radiation impedance [2]. As a result, long-duration pulse-compression waveforms are employed in pulsed active sonars to deal with these constraints. The pulse-compression property can be achieved by modulations of amplitude, phase, or frequency. The most popular pulse-compression waveform is the linear frequency-modulated (LFM) waveform, which achieves both long pulse duration and wide bandwidth.

Nowadays, the matched filter is utilized in most active sonar systems. It estimates the range and velocity of targets through the correlation between replicas and echoes. If there is no relative motion between the targets and the sonar system, the replicas of the matched filter will have an accurate match with echoes from the target. Once a relative motion emerges, a mismatch will occur due to the Doppler effect, which is referred to as the ‘range-Doppler ambiguity’ problem and frequently occurs in traditional continuous wave (CW) or LFM waveforms of pulse sonar systems. It was indicated that the CW is appropriate for detecting high-speed targets in a reverberation environment, while the LFM is suitable for detecting low-speed or stationary targets [3]. Hence, it was suggested to combine two different waveforms to improve the detection performance of active sonars. As a result, the continuous transmission of composite pulse trains was developed [4]. Compared with traditional pulse waveforms, the continuous pulse trains can effectively reduce the range-Doppler ambiguity of detection and result in higher resolutions of range and velocity. Higher transmission and signal-noise ratio gains can also be achieved due to the longer correlation durations. Recently, several modulated pulse train waveforms have been developed. Costas first found that the specific frequency-modulated signals exhibit high range and velocity resolutions, as well as high reverberation suppression performance [5]. Pechnold proposed using the Costas sequence to improve the performance of pulsed active sonars [6]. Hickman presented the LFM-Costas pulse train, which was the LFM pulses coded by the Costas sequence [7]. It was pointed out that this waveform employed the periodic property of the Costas sequence to achieve continuous tracking and high detection resolution. DeFerrari proposed a different approach to design continuous active sonar signals based on the maximal-length sequence [8]. The results showed that the maximal-length sequence provided the better detecting performance in reverberation and the reduction of the direct blast. Hague researched a generalized sinusoidal frequency-modulated (GSFM) waveform [9]. This waveform was designed to have low cross-correlations, which can reduce the interference from the direct blast and improve the efficiency of echo signal processing. Liang devised a method of processing multistatic active sonar signals using the improved PeCan phase-modulated waveform as the transmitted signal [10]. Lourey further studied the hopped frequency-modulated waveform, which exhibited improved range resolution compared to LFM at the cost of a deteriorative interference level [11]. Wang proposed a method of estimating range and velocity of targets through a continuous transmission of composite hyperbolic frequency-modulated signals [3]

For an active sonar system, the performance of transmitted signals can be evaluated by the ambiguity function (AF). It depicts the response of a match filter with different time delays and Doppler shifts [12]. Furthermore, the reverberation suppression performance can be estimated directly from the zero-time-delay cut of AF, which is known as Q-function [6]. Mathematically, the AF expression is composed of time delays, Doppler shifts, and the characteristic parameters of the waveform, such as bandwidth, carrier frequency, duration, etc. [5]. Moreover, the AF expression can be visualized as a three-dimensional shape on the time delay-velocity pedestal. The width of the mainlobe and the sidelobe levels are the main characteristics of the AF shape. The width of the mainlobe is inversely proportional to the resolution, distinguishing closely-spaced targets in range or velocity. The sidelobe levels, which represent the sound intensities of sidelobes, can evaluate the ability to detect targets in the reverberation environment. The higher sidelobe levels imply that the target detection is interfered by the reverberation more seriously. In particular, some active sonar waveforms show the AF shape that features a distinct mainlobe at the origin of the time delay–velocity pedestal and sidelobes at the rest of the pedestal evenly, which is known as the ‘thumbtack’ AF shape [13]. Furthermore, this ideal ‘thumbtack’ AF shape might be reached by optimizing specific parameters in the AF expression. Several methods have been developed for optimizations of polyphase sequences in radar and sonar systems. Liu utilized Tabu search to design orthogonal phase-modulated waveforms for active sonar systems [14]. Sharma optimized four-phase orthogonal sequences for MIMO radar systems by means of the alternate sequence generation method [15]. However, frequency-modulated signals have lower degrees of freedom than that of phase-modulated signals. Additionally, the optimization objectives of these two kinds of signals are totally different. Therefore, the aforementioned methods are not appropriate for the research on frequency-modulated waveforms. Zhao proposed another approach for the optimal sidelobe design of the hopped-frequency waveform with the adaptive gradient search [16]. Moreover, Mehany and Wang both used the genetic algorithm (GA) to optimize orthogonal waveforms for radar systems [17,18].

This paper focuses on the optimal waveform design of several frequency-modulated pulse trains with GA. The first waveform to be optimized is the LFM-Costas pulse train. Through the modulation of Costas sequence, the range-Doppler ambiguity problem of LFM is alleviated, but the Doppler ambiguity problem still exists, which is what we are concerned about in this paper. The second pulse train is the generalized sinusoidal frequency-modulated (GSFM) waveform, which features the thumbtack AF mainlobe. However, there are still relatively high sidelobe levels in the AF under present parameter settings [9]. Thus, the optimal waveform design of the GSFM pulse train needs to be further researched. 

The rest of this paper is organized as follows. Section 2 describes the quantitative AF expressions of continuous pulse trains, which are essential for our research. The optimization model of frequency-modulated pulse trains is given in Section 3, and the optimized objectives include AF shape and the zero-velocity cut of AF. In addition, the correlations between the sub-pulses of the GSFM pulse train are also the optimized objective. Evaluation criteria for optimizing the frequency-modulated pulse trains are then formulated, which can be minimized using GA. The experimental simulations and analysis are provided in Section 4, and the optimal parameters of the LFM-Costas and GSFM pulse trains are obtained. The AF properties of two optimized pulse trains are analyzed, and their reverberation suppression performance is also discussed by means of Q-function. Finally, Section 5 presents the conclusions of this paper.

## 2. AF Quantitative Expression of Frequency-Modulated Pulse Train

The AF properties of the pulse train are demonstrated by its AF shape, which is related to the AF quantitative expression. Due to the indeterminacy of the quantitative expressions, the AF shape is alterable so that some specific AF properties can be obtained. Then, it is possible to improve both the AF shape and the properties of a specific pulse train through the modulation of its AF expression. In this paper, an optimization model is formed to improve the required AF properties. The model is primarily regulated by the AF quantitative expression. For this reason, it is necessary to derive the quantitative expressions before we construct the model. The AF expressions of the basic Costas sequence, LFM pulse, and LFM-Costas pulse train have been outlined by [5] and [19], respectively, while the expression of the GSFM pulse train remains to be derived. Hence, we present an AF expression adaptable to all kinds of frequency-modulated pulse trains involving the LFM-Costas and GSFM, which are optimized in the next section.

A continuous pulse train consisting of N pulses can be expressed as follows:

(1) $$u\left(t\right)=\frac{1}{\sqrt{Nt_{p}}}\sum_{n=0}^{N-1}u_{n}\left(t-nt_{p}\right)$$

where $t_{p}$ is the duration of sub-pulses, and $u_{n}\left(t\right)$ represents the *n*th sub-pulse of the train, which can be written as:

(2) $$u_{n}\left(t\right)=\frac{\mathrm{rect}\left(t\right)}{\sqrt{t_{p}}}A_{n}\left(t\right)e^{j2\pi f_{n}t},0\leq t\leq t_{p}$$

where rect(t) is the rectangular function, $f_{n}$ is the hopped frequency of the *n*th sub-pulse, and $A_{n}\left(t\right)$ is the complex envelope of $u_{n}\left(t\right)$. Assuming that the target velocity is much lower than the sound speed and the ratio of bandwidth with carrier frequency $B/2f_{0}$ is low (≤0.1), which means that the pulse train can be simplified in the form of narrowband, then the narrowband cross-ambiguity function (NCAF) of different sub-pulses $u_{n}\left(t\right)$ and $u_{m}\left(t\right)$ can be expressed as [20]:

(3) $$\begin{array}{ll} \chi_{n,m}\left(\tau ,\varphi \right) & =\frac{1}{t_{p}}\int_{-\infty }^{+\infty }u_{n}\left(t\right)u_{m}^{*}\left(t-\tau \right)e^{j2\pi \varphi t}dt\\ & =\frac{1}{t_{p}}e^{-j2\pi f_{m}\tau }\int_{-\infty }^{+\infty }A_{n}\left(t\right)A_{m}^{*}\left(t-\tau \right)e^{j2\pi \left(\varphi -\left(f_{n}-f_{m}\right)\right)t}dt\\ & =\frac{1}{t_{p}}e^{-j2\pi f_{m}\tau }\chi_{A}\left(\tau ,\varphi -\left(f_{n}-f_{m}\right)\right) \end{array}$$

where $\tau \left(\left|\tau \right|\leq t_{p}\right)$ is the time delay and $\varphi =\frac{2v}{c}f_{0}$ is the Doppler shift of echoes. $\chi_{A}\left(\tau ,\varphi \right)$ is defined as the AF of the complex envelopes $A_{n}\left(t\right)$. The narrowband auto-ambiguity function (NAAF) $\chi_{n,n}\left(\tau ,\varphi \right)$ can be expressed by replacing the subscript m with n in Equation (3). According to [5], the AF expression of continuous pulse train can be written as:

(4) $$\chi \left(\tau ,\varphi \right)=\frac{1}{N}\sum_{n=0}^{N-1}e^{j2\pi n\varphi t_{p}}\left[\chi_{n,n}\left(\tau ,\varphi \right)+\sum_{\begin{array}{l} m=0\\ m\neq n \end{array}}^{N-1}\chi_{n,m}\left(\tau -\left(n-m\right)t_{p},\varphi \right)\right]$$

then, substituting $\chi_{n,m}\left(\tau ,\varphi \right)$ and $\chi_{n,n}\left(\tau ,\varphi \right)$ into Equation (4), the AF expression of the frequency-modulated pulse train can be rewritten as:

(5) $$\chi \left(\tau ,\varphi \right)=\frac{1}{Nt_{p}}\sum_{n=0}^{N-1}e^{j2\pi n\varphi t_{p}}[\chi_{A}\left(\tau ,\varphi \right)e^{-j2\pi f_{n}\tau }+\sum_{\begin{array}{l} m=0\\ m\neq n \end{array}}^{N-1}\chi_{A}\left(\tau -\left(n-m\right)t_{p},\varphi -\left(f_{n}-f_{m}\right)\right)e^{-j2\pi f_{m}\left(\tau -\left(n-m\right)t_{p}\right)}]$$

According to Equation (5), the AF quantitative expressions of all kinds of frequency-modulated pulse trains are represented by the AF expressions of the complex envelopes $A_{n}\left(t\right)$ with different time delays and frequency shifts. As aforementioned, some AF expressions of pulses’ complex envelopes have been given out. In particular, the *n*th sub-pulse of the LFM-Costas pulse train can be written as:

(6) $$u_{n}\left(t\right)=\frac{\mathrm{rect}\left(t\right)}{\sqrt{t_{p}}}e^{j2\pi \left(f_{0}t+\frac{1}{2}\mu t^{2}\right)}e^{j2\pi f_{n}t}$$

which has the carrier frequency $f_{0}$ and the linear modulation index μ. $f_{n}=a_{n}\Delta f$ ($\left\{a_{n}\right\}$ is the Costas sequence and Δf is the frequency separation. Here, the complex envelope $A_{n}\left(t\right)$ is defined as $A_{n}\left(t\right)=\exp\left(j2\pi \left(f_{0}t+\frac{1}{2}\mu t^{2}\right)\right)$. According to the conclusion in [19], the AF expression of the complex envelopes $A_{n}\left(t\right)$ has been derived as:

(7) $$\chi_{A}\left(\tau ,\varphi \right)=\left|\left(t_{p}-\left|\tau \right|\right)\frac{\sin\left(\pi \left(\varphi t_{p}-\tau B\right)\left(1-\left|\tau \right|/t_{p}\right)\right)}{\pi \left(\varphi t_{p}-\tau B\right)\left(1-\left|\tau \right|/t_{p}\right)}e^{j\pi \varphi \tau }\right|$$

**Figure 1:**
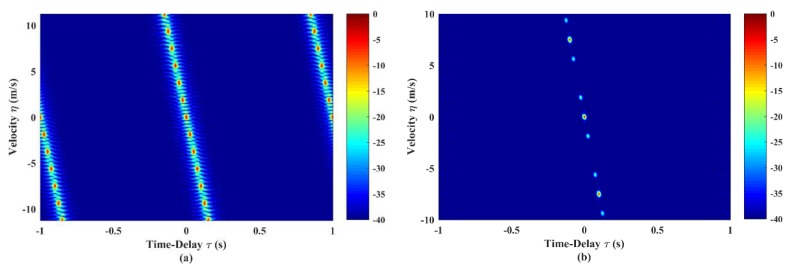
Ambiguity function (AF) shapes of (**a**) linear frequency-modulated (LFM) pulse train and (**b**) LFM-Costas pulse train. They both have N=30, $T=30t_{p}=30s$, $f_{0}=2\mathrm{KHz}$ and B=200Hz. The LFM-Costas uses a 30-code Costas sequence and Δf=10Hz.

Therefore, substituting $\chi_{A}\left(\tau ,\varphi \right)$ back into Equation (5), we can obtain the AF expression of LFM-Costas. Figure 1a,b shows the AF shapes of the LFM and LFM-Costas pulse trains, respectively. In Figure 1a, the LFM pulse train exhibits severe Doppler ambiguity and periodic repetitions of sidelobes. Figure 1b demonstrates that the Doppler ambiguity problem of the LFM pulse train has been significantly improved. However, ambiguity along the velocity dimension still exists in LFM-Costas.

Analogously, the closed-form AF of the GSFM pulse train can be achieved as the above derivation of LFM-Costas. The expression of the GSFM sub-pulse can be written as:

(8) $$u_{n}\left(t\right)=\frac{\mathrm{rect}\left(t\right)}{\sqrt{t_{p}}}e^{j\frac{\beta }{t^{\rho_{n}-1}}\sin\left(\frac{2\pi \alpha_{n}t^{\rho_{n}}}{\rho_{n}}\right)}e^{j2\pi f_{0}t}$$

where $\beta =B/2f_{M}$ is the modulation index, $f_{M}$ is the modulation frequency, and B is the bandwidth. ρ is a unitless parameter that must be greater than or equal to 1, while α is a frequency modulation term with units $s^{-\rho }$ [9]. This paper focus on the GSFM pulse with even-symmetric instantaneous-frequency, which replaces $t^{\rho_{n}}$ in Equation (8) with ${\left|t\right|}^{\rho_{n}}$. This kind of GSFM pulse has the same performance as the normal kind of Equation (8) and can simplify the mathematical processing. According to the research in [20], the AF expression of an even-symmetric GSFM pulse can be expressed as:

(9) $$\chi_{A}\left(\tau ,\varphi \right)=\left(\frac{t_{p}-\left|\tau \right|}{t_{p}}\right)\times \left|\sum_{n=-\infty }^{+\infty }I_{n}\left\{2jF{\tilde{a}}_{m}\sin\left(\frac{\pi m\tau }{t_{p}}\right)\right\}\times \sin c\left[\pi \left(\frac{n}{t_{p}}+\varphi \right)\left(t_{p}-\left|\tau \right|\right)\right]\right|$$

where ${\tilde{a}}_{m}=\frac{2}{mt_{p}}\int_{0}^{T}f_{\mathrm{GSFM}}\left(t\right)\cos\left(\frac{m\pi t}{t_{p}}\right)dt\left(m=0,1,\cdots \right)$ are the Fourier coefficients of instantaneous frequency $f_{\mathrm{GSFM}}\left(t\right)$, and $I_{n}\left\{2jF{\tilde{a}}_{m}\sin\left(\frac{-\pi m\tau }{t_{p}}\right)\right\}$ is the infinite-dimension generalized Bessel function (GBF) of the modified form [21].

Substituting Equation (9) into the expressions of NAAF $\chi_{n,n}$ and NCAF $\chi_{n,m}$, then according to Equation (5), the AF expression of GSFM pulse train can be expressed as the following:

(10) $$\chi \left(\tau ,\varphi \right)=\frac{1}{Nt_{p}}\sum_{n=0}^{N-1}e^{j2\pi n\varphi t_{p}}[\chi_{A}\left(\tau ,\varphi \right)e^{-j2\pi f_{0}\tau }+\sum_{\begin{array}{l} m=0\\ m\neq n \end{array}}^{N-1}\chi_{A}\left(\tau -\left(n-m\right)t_{p},\varphi \right)e^{-j2\pi f_{0}\left(\tau -\left(n-m\right)t_{p}\right)}]$$

**Figure 2:**
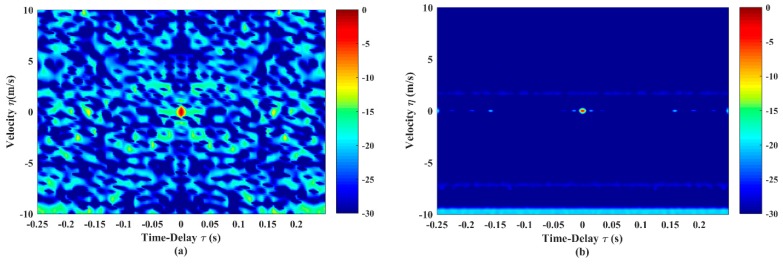
The AF shapes of (**a**) single generalized sinusoidal frequency-modulated (GSFM) pulse and (**b**) continuous pulse train (N=8). The duration of the sub-pulse $t_{p}=0.25s$, carrier frequency $f_{0}=2\mathrm{KHz}$, and bandwidth B=400Hz. All sub-pulses have the identical characteristic parameters $\alpha_{n}=160,\rho_{n}=2,n=1,\ldots ,N$.

The quantitative AF expression of the GSFM pulse train is derived through Equation (10). Figure 2 shows the AF shape of the GSFM single pulse and the corresponding pulse train with size N=8. It is worth noting that the $t_{p}$ of GSFM is different from LFM and LFM-Costas ($t_{p}=1s$). The reason is that the longer-duration GSFM pulse ($t_{p}>0.25s$) yields the worst AF performance. Our previous research in [22] shows that the GSFM pulse with $t_{p}=0.25s$ have the best AF and correlation properties. Compared with Figure 1, the AF shape of GSFM pulse in Figure 2a clearly shows the thumbtack mainlobe and there is almost no evidence of a range-Doppler ambiguity problem. However, the sidelobe levels of the single pulse are still high. The AF shape in Figure 2b features a narrower mainlobe and lower sidelobes than Figure 2a in both time delay and velocity. This demonstrates that the increasing duration caused by continuous transmission of pulses can sharpen the mainlobe and reduce the sidelobe levels in AF, which improves the target detection in a reverberation environment. However, some sidelobes still exist, especially along the velocity −10m/s.

## 3. Optimization Model of Frequency-Modulated Pulse Trains

In Section 2, we found that there still exists Doppler ambiguity of AF in LFM-Costas. Additionally, the AF of GSFM pulse train has some sidelobe interference along the dimension of velocity. As a result, it would be useful to construct an optimization model to improve the detection performance of the frequency-modulated pulse train. There are three objectives to be optimized. Firstly, the optimization of the Auto-Correlation Function (ACF) sidelobe area aims to generate the ACF curve with a sharper mainlobe and lower average sidelobe levels, which represents a higher range resolution of the pulse train. Secondly, the optimization of AF sidelobe levels focuses on the improvement of the ability to distinguish the true target in reverberation. Thirdly, the optimization of correlation properties aims to reduce the correlations between sub-pulses, which needs to be considered for GSFM pulse train to eliminate the direct blast. All of the three objectives are expressed by the following fitness functions, which are closely connected with parameters of the pulse train expressions. Minimizing these objectives with GA, key parameters and the corresponding optimal frequency-modulated pulse train are achieved.

### 3.1. Optimization of the ACF Sidelobe Area

In this paper, the ACF is defined as the zero-velocity cut of AF. To some extent, it reflects the correlations between the replicas and echoes. Moreover, as Hague suggested in [13], the ACF is of great significance to the evaluation of the range resolution. Specifically, the ACF expression can be derived if we substitute the Doppler shift φ=0 into the AF quantitative expression. As an example, the ACF expression of LFM-Costas can be written as:

(11) $$R\left(\tau \right)\equiv \chi \left(\tau ,0\right)=\frac{1}{Nt_{p}}\sum_{n=0}^{N-1}[\chi_{p}\left(\tau ,0\right)e^{-j2\pi f_{n}\tau }+\sum_{\begin{array}{l} m=0\\ m\neq n \end{array}}^{N-1}\chi_{p}\left(\tau -\left(n-m\right)t_{p},\left(f_{m}-f_{n}\right)\right)e^{-j2\pi f_{m}\left(\tau -\left(n-m\right)t_{p}\right)}]$$

Here, $\chi_{p}\left(\tau ,0\right)$ is the ACF of the LFM pulse, which can be derived from Equation (7). It is desirable that the optimal ACF should have the sharper mainlobe and lower sidelobes. The sidelobes are expected to be as low as possible, particularly in the region near the mainlobe. Meanwhile, the mainlobe shape should be maintained so that the range resolution does not get worse. Hence, the area enclosed by the sidelobes is identified as the optimization objective of ACF, which can be represented as the following fitness function:

(12) $$F_{1}=\underset{C}{\min}\left[\int_{\Omega_{\tau }}\left|R\left(\tau \right)\right|d\tau \right]$$

where $\Omega_{\tau }$ is the optimized ranges of ACF sidelobes on time delays. The set C represents optimized parameters of frequency-modulated pulse trains.

### 3.2. Optimization of AF Sidelobe Levels

As stated previously, the optimization of AF sidelobe levels aims to improve the accuracy of detecting targets in the reverberation. The AF sidelobe levels should be minimized in the interested region, while the width of the mainlobe should be maintained in either range or velocity. According to [5], AF sidelobes are only dependent on the second sum term of expressions in Equation (5), which can be rewritten as follows:

(13) $$\chi_{\mathrm{part}2}\left(\tau ,\varphi \right)=\sum_{\begin{array}{l} m=0\\ m\neq n \end{array}}^{N-1}\chi_{p}\left(\tau -\left(n-m\right)t_{p},\varphi -\left(f_{n}-f_{m}\right)\right)e^{-j2\pi f_{0}\left(\tau -\left(n-m\right)t_{p}\right)}$$

In order to reduce the computational burden, we can concentrate on Equation (13) for the optimization of AF sidelobe levels. Since the optimization object focuses on the minimization of AF sidelobe levels (height) in the interested region (area), we can represent this object with the integral of height and the area, which is the volume of sidelobes. As a result, the optimization fitness can be stated as:

(14) $$F_{2}=\underset{C}{\min}\left[\iint_{\Omega_{\tau ,\varphi }}\left|\chi_{\mathrm{part}2}\left(\tau ,\varphi \right)\right|d\tau d\varphi \right]$$

where $\Omega_{\tau ,\varphi }$ is the optimized region of AF sidelobes in the time delay-velocity pedestal.

### 3.3. Optimization of the Correlation Properties

The LFM-Costas sub-pulses already have low cross-correlation properties due to the orthogonality of the Costas sequence. On the other hand, the correlation properties of the GSFM pulse train remain to be optimized because of its indeterminate parameters $\left\{\alpha_{n}\right\},\left\{\rho_{n}\right\}$. Hence, the optimization objective in this sub-section plans to minimize the autocorrelation sidelobe levels of each sub-pulse and the average cross-correlation levels between sub-pulses in the GSFM pulse train.

Considering a GSFM pulse train with N sub-pulses $u_{n}\left(t\right)$, the length of each pulse is L. The aperiodic correlation can be defined as [17]:

(15) $$R\left(p,q,k\right)=\{\begin{array}{c} \frac{1}{L}\sum_{l=1}^{L-k}u_{p}\left(l\right)u_{q}^{*}\left(l+k\right),0\leq k<L\\ \frac{1}{L}\sum_{l=-k+1}^{L}u_{p}\left(l\right)u_{q}^{*}\left(l+k\right),-L<k<0 \end{array}$$

where p,q=1,2,⋯,N, *k* is the discrete time index. When p=q, R(p,q,k) is the aperiodic auto-correlation function of pulse $u_{p}$; conversely, R(p,q,k) is the cross-correlation function between $u_{p}$ and $u_{q}$ when p≠q. Taking the conjugate-symmetry into account, the sidelobe of auto-correlation can be expressed as:

(16) $$A\left(p,k\right)={\left[R\left(p,p,1\right),R\left(p,p,2\right),\cdots ,R\left(p,p,L-1\right)\right]}^{T},k=1,2,\cdots ,L-1$$

Let us define A(p,0)=R(p,p,0) as the mainlobe of auto-correlation. Moreover, the cross-correlation between two sequences is defined as:

(17) $$C\left(p,q\right)={\left[R\left(p,q,-L+1\right),R\left(p,q,-L+2\right),\cdots R\left(p,q,L-1\right)\right]}^{T}$$

Consequently, the normalized levels of the auto-correlation sidelobe (ACS) peak and cross-correlation (CC) peak can be expressed as:

(18) $$\left\{ \begin{array}{l} \mathrm{ACS}\_\mathrm{peak}=\sum_{p=1}^{N}\underset{k=1,2,\cdots ,L-1}{\max}\left|\frac{A\left(p,k\right)}{A\left(p,0\right)}\right|\\ \mathrm{CC}\_\mathrm{peak}=\sum_{p=1}^{N}\sum_{q=p+1}^{N}\max\left|\frac{C\left(p,q\right)}{\max\left|A\left(p,0\right)\right|}\right| \end{array}\right.$$

Both ACS_peak and CC_peak should be minimized so that the auto-correlation and cross-correlation properties of the GSFM pulse train can be optimized. Since the duration $t_{p}$, carrier frequency $f_{0}$ of sub-pulses are assumed to be identical, the difference between each pulse depends only on $\left\{\alpha_{n}\right\}$, $\left\{\rho_{n}\right\}$. Accordingly, the fitness function to minimize (18) can be expressed as:

(19) $$F_{3}=\underset{C}{\min}\left(\mathrm{ACS}\_\mathrm{peak}+\mathrm{CC}\_\mathrm{peak}\right)$$

Here, the set C specifically represents $\left\{\alpha_{n}\right\}$ and $\left\{\rho_{n}\right\}$ of the GSFM pulse train. In order to not only minimize the sidelobe peak but also keep the average sidelobe levels low, the total auto-correlation sidelobe energy (ACS_E) and cross-correlation energy (CC_E) should also be taken into consideration. They are defined as follows:

(20) $$\left\{ \begin{array}{l} \mathrm{ACS}\_E=\sum_{k=-\left(L-1\right)}^{L-1}{\left|A\left(p,k\right)\right|}^{2}-{\left|A\left(p,0\right)\right|}^{2}\\ \mathrm{CC}\_E=\sum_{k=-\left(L-1\right)}^{L-1}{\left|C\left(p,q,k\right)\right|}^{2} \end{array}\right.$$

Thus, the optimization of sidelobe energy can be expressed as:

(21) $$F_{4}=\underset{C}{\min}\left(\mathrm{ACS}\_E+\mathrm{CC}\_E\right)$$

In Equation (21), it is worth noticing that the optimization only considers minimizing the sum form of ACS_E and CC_E rather that minimizing them respectively. The reason is that this reaches a compromise between the auto-correlation and the cross-correlation. The same reason is applied for Equation (19).

### 3.4. Optimization Model with the Genetic Algorithm

In this paper, we discuss an optimization of multiple objectives under a certain constraint condition. A robust algorithm with global optimum searching capability is necessary. So we utilize the genetic algorithm to construct the model in that it is an adaptive and robust method which can evolve to globally optimal parameters of pulse trains. For the LFM-Costas pulse train, the model should include optimizations of both ACF and AF sidelobe levels, which are evaluated by fitness functions $F_{1}$ and $F_{2}$. On the contrary, $F_{3}$ and $F_{4}$ are overlooked in that the Costas sequence determines the orthogonality between sub-pulses. The parameters to be optimized are the linear modulation index μ and frequency spacing Δf. For the GSFM pulse train, fitness functions $F_{1}∼F_{4}$ should be totally taken into account. The parameters to be optimized is the frequency modulation term $\alpha_{n}$ and $\rho_{n}$. As a result, the optimization model of two pulse trains can be constructed, respectively, as follows:

(22) $$F_{\mathrm{Costas}}\left(\mu ,\Delta f\right)=w_{1}\cdot F_{1}+w_{2}\cdot F_{2}$$

(23) $$F_{\mathrm{GSFM}}\left(\alpha_{n},\rho_{n}\right)=w_{1}\cdot F_{1}+w_{2}\cdot F_{2}+w_{3}\cdot F_{3}+w_{4}\cdot F_{4}$$

where $W=\left[w_{1}{,w}_{2}{,w}_{3}{,w}_{4}\right]$ are weighting coefficients. Through the GA, optimal pulse trains and the corresponding parameters can be obtained by minimizing (22) or (23).

Moreover, it is worth noticing that the units of the fitness functions are quite different and represent area ($F_{1}$), volume ($F_{2}$), and energy ($F_{3},F_{4}$), respectively. Hence, the traditional weighting coefficient settings result in immoderate weight between each fitness function. In this model, we implement an average adaptive weight approach in order to achieve global searching and improve the population diversity of multi-objective optimization [23]. 

For an optimization problem with n objectives, we define the initial weighting coefficients as $w_{j}^{\left(0\right)}=1/n,j=1,\cdots ,n$. The mean value of each fitness function in the *p*th generation $F_{1}^{\left(p\right)}\left(x\right),\cdots ,F_{n}^{\left(p\right)}\left(x\right)$ can be written as follows:

(24) $$\bar{F_{k}^{\left(p\right)}}=\frac{1}{m}\sum_{i=1}^{m}F_{k}^{\left(p\right)}\left(x_{i}\right),k=1,2,\cdots ,n$$

where $\left\{x_{i}\right\},i=1,\cdots ,m$ are chromosomes in the *p*th generation. According to the approach in [23], the weighting coefficients vary in each generation:

(25) $$w_{i}^{\left(p+1\right)}=\frac{\frac{1}{n}\sum_{k=1}^{n}\bar{F_{k}^{\left(p\right)}}}{\bar{F_{k}^{\left(p\right)}}}w_{i}^{\left(p\right)}$$

Then the overall objective function in the (*p* + 1) th generation can be expressed as follows:

(26) $$Eval^{\left(p+1\right)}=\sum_{j=1}^{n}w_{i}^{\left(p+1\right)}eval_{j}^{\left(p+1\right)}$$

Through the above-mentioned approach, the weighting coefficients are adjusted effectively in each generation of GA. As a result, all of the objectives of the model can be optimized evenly.

## 4. Optimization Results and Analysis

In this section, several simulations were applied to analyze the performance of the developed optimization model. The settings of the GA are as follows: Population items are 100, the probability of crossover is 0.7, and the probability of mutation is 0.1. The algorithm iterates over 200 generations. All the simulations are performed on a PC with a 3.50 GHz i7–3770K CPU and 4 GB RAM.

### 4.1. LF-Costas Pulse Train

**Figure 3:**
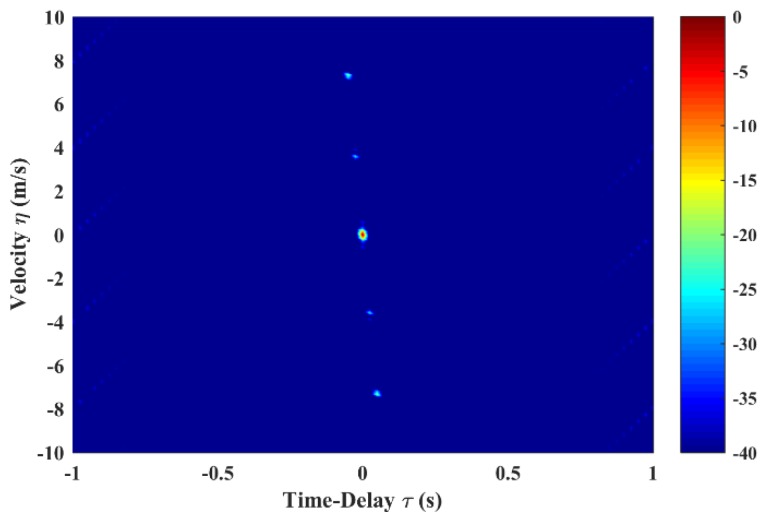
AF shapes of LFM-Costas pulse train after optimization. The optimized parameters are $\mu_{t}=391.994\mathrm{Hz}/s$ and $\Delta f_{t}=10.621\mathrm{Hz}$. Other settings are the same as Figure 1b.

The simulation in this sub-section uses 30 codes LFM-Costas, each sub-pulse has duration $t_{p}=1$s. The initial pulse train has parameters of $B_{0}=200\mathrm{Hz},\mu_{0}=200\mathrm{Hz}/s,\Delta f_{0}=10\mathrm{Hz}$ (see Figure 1b). Two parameters μ and Δf are optimized, respectively. Figure 3 shows the AF shapes of LFM-Costas pulse train after optimization. In comparison with the initial AF shapes in Figure 1b, the levels of instinct sidelobe peaks are improved to be too low to mask the mainlobe, while the width of the mainlobe is not changed, which makes it more accurate to estimate the velocity of targets. 

**Figure 4:**
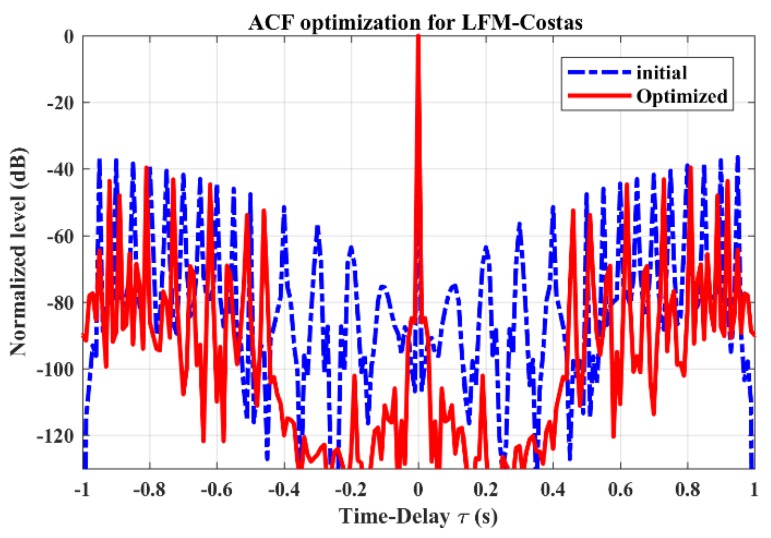
The Auto-Correlation Function (ACF) optimization for LFM-Costas pulse train. The blue dotted line represents the initial LFM-Costas in Figure 1b. The solid red line represents the optimized LFM-Costas in Figure 3. Here, the ACF levels are normalized and expressed in dB as $10\log_{10}\left\{R\left(\tau \right)/\underset{\tau }{\max}\left[R\left(\tau \right)\right]\right\}$.

The ACF of the LFM-Costas pulse train is shown in Figure 4. Compared with the initial pulse train, the optimized pulse train demonstrates lower sidelobe levels, especially in the region near the mainlobe. This improves the ability of LFM-Costas to detect a weaker target in a reverberation environment.

The sidelobe levels of the LFM-Costas pulse train are presented in Table 1. The average sidelobe level of the optimized AF is slightly better (column 1). On the other hand, the peak sidelobe level achieved the greatest reduction, over 17 dB (column 2). The optimized average and peak level of ACF sidelobes in column 3 and 4 decrease substantially. The reduction of the sidelobe levels confirms the validity of the optimization model on LFM-Costas. 

### 4.2. GSFM Pulse Train

**Figure 5:**
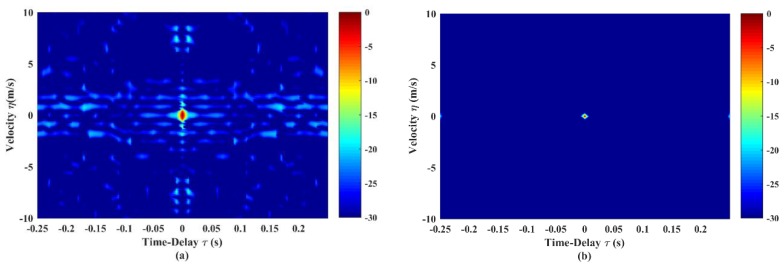
AF shapes of (**a**) GSFM pulse and (**b**) continuous pulse train (N=8) after optimization. The GSFM pulse in Figure 5a has the optimized parameters α=1966,ρ=2.21, and it composes the GSFM pulse train in Figure 5b.

In this sub-section, we demonstrate the validity of the optimization model on GSFM. A pulse train with size of N=8 is optimized in the simulations. All GSFM sub-pulses are the even symmetric style. The initial settings shown in Figure 2 are totally the same with those in [9,13,20]. The optimized AF shape of the GSFM pulse and the corresponding pulse train are shown in Figure 5. Compared with Figure 2a, the AF sidelobes in Figure 5a are reduced dramatically. The average sidelobe level is reduced from –29.73 to –39.68 dB and the peak sidelobe level is reduced from –10.7 to –18.09 dB. Furthermore, the thumbtack mainlobe tends to be more distinct. In Figure 5b, the width of the mainlobe decreases in both dimension of time delay and velocity. Sidelobes in Figure 2b have also vanished completely.

**Figure 6:**
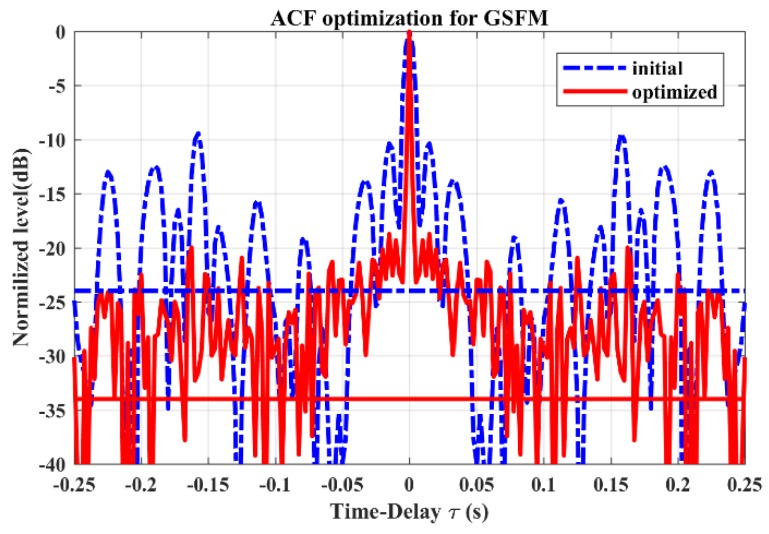
The ACF optimization for GSFM pulse train. The horizontal dotted blue line and solid red line, respectively, represent the average ACF sidelobe level before and after optimization.

Figure 6 shows the ACF optimization results. After the optimization, there is an obvious reduction in the region near the mainlobe, which suggests a higher resolution in range (time delay). A decline of average sidelobe levels (from –23.7 to –28.7 dB) can also be observed through the difference between the horizontal dotted blue line and the solid red line. Meanwhile, the peak sidelobe levels are reduced from –9.36 to –18.72 dB.

**Figure 7:**
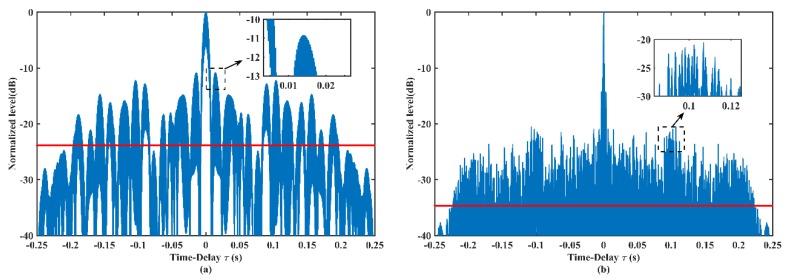
Autocorrelations of (**a**) the initial GSFM pulse and (**b**) the optimized GSFM pulse. Their parameters are same as Figure 2a and Figure 5a, respectively. The solid red lines represent the average sidelobe levels.

Figure 7 shows the autocorrelation curves of the pulse train before and after optimization. Figure 7a presents the autocorrelation of initial GSFM pulse, while Figure 7b presents the autocorrelation of the optimized pulse. The solid red lines represent the average levels of sidelobes, which are reduced from –24 dB (Figure 7a) to –34 dB (Figure 7b). The peak sidelobe (in the magnifying plots) is lowered from –11 to over –20 dB.

**Figure 8:**
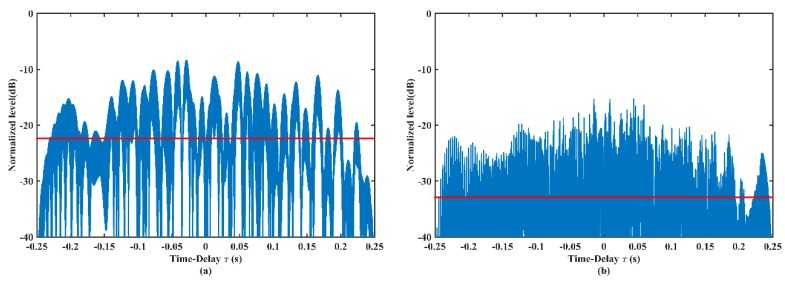
Cross-correlations of (**a**) the initial GSFM pulses and (**b**) the optimized GSFM pulses. (**a**) is the cross-correlation between initial pulses with the normal instantaneous-frequency and the even-symmetric instantaneous-frequency, while (**b**) is the cross-correlation between pulse 1 and 2 with $\alpha_{1}=1966,\rho_{1}=2.21$ and $\alpha_{2}=1877,\rho_{2}=2.18$.

Figure 8 demonstrates the cross-correlation curves of pulse trains before and after optimization. Figure 8a presents the cross-correlation between two initial sub-pulses. The average sidelobe level (the solid red line) is –22.38 dB and the peak sidelobe level is –8.36 dB. Figure 8b shows the cross-correlation between two optimized sub-pulses. The average sidelobe level is –32.89 dB and the peak sidelobe level is –16.45 dB. Compared with Figure 8a, both the average level and the peak level in Figure 8b have obvious reductions. The lower cross-correlation level implies the better performance to mitigate interference from the direct blast. 

### 4.3. Q-Function Performance

Reverberation has significant influence on the detection performance of active sonar systems, particularly in shallow water environments. As a result, the detecting performance of transmitted signals in reverberation must be taken into account. This performance is usually evaluated by the Q-function, which shows the detection ability of signals on targets with different velocity in a reverberation environment [6]. In this paper, we also use this method to estimate the reverberation suppression performance of optimized pulse trains. The Q-function is known to be relative to the zero-time-delay cut of AF and is always written as:

(27) $$Q\left(\varphi \right)=\int_{-\infty }^{+\infty }{\left|\chi \left(\tau ,\varphi \right)\right|}^{2}d\tau$$

**Figure 9:**
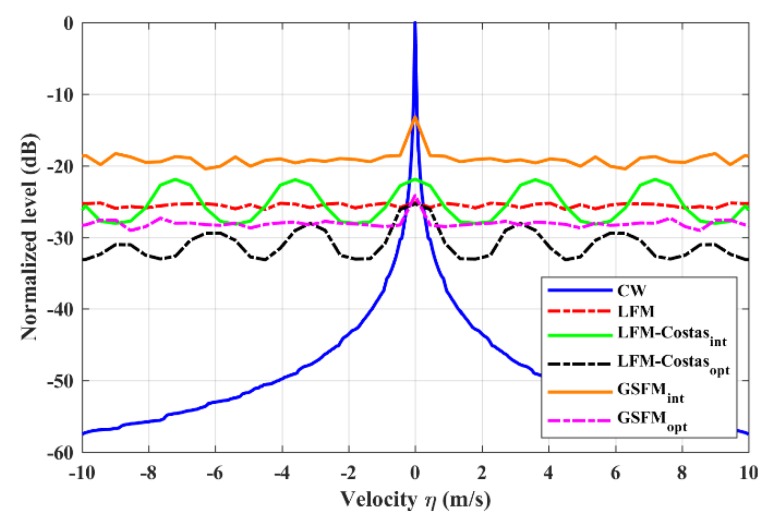
Q-functions of several continuous pulse trains.

Figure 9 presents the Q-functions of six continuous pulse trains. The initial and optimized LFM-Costas and GSFM pulse trains are compared with the most widely used continuous signals, namely CW and LFM. All six waveforms have the same total duration as T=30s. The LFM pulse train has N=30 and the same settings as Figure 1a. The initial and optimized LFM-Costas have the same settings as Figure 1b and Figure 3, respectively. The settings of initial and optimized GSFM sub-pulses are the same as Figure 2a and Figure 5a, respectively, which means the sub-pulse duration is $t_{p}=0.25s$. Hence, the sub-pulse number of GSFM is $N=T/t_{p}=120$. As Figure 9 shows, CW performs poorly in a reverberation environment for detecting stationary targets. However, the reverberation levels decline rapidly as the velocities become greater, which shows that CW is appropriate for detecting high-speed targets in a reverberation environment. The initial GSFM obviously has the highest reverberation levels in the non-zero-velocity zone. However, the curves in this zone are nearly flat, which shows the Doppler tolerance of the GSFM. The optimized GSFM provides a better reverberation suppression performance and maintains the Doppler insensitivity similar with the LFM pulse train. The Q-function curve of initial LFM-Costas has five periodic peaks in 0m/s, ±3.6m/s and ±7.2m/s which achieve identical levels. After the optimization, the LFM-Costas gets a reduction of the reverberation levels in all the velocities. The peaks exist in 0m/s, ±3.2m/s, ±6m/s and ±8.8m/s, but these in the non-zero-velocity zone are no longer as high as the peak in 0m/s. 

## 5. Conclusions

This paper discussed the optimal waveform design for two kinds of frequency-modulated continuous pulse train. Based on the quantitative AF expression, the detection performance of pulse trains was analyzed, showing that there is the Doppler ambiguity in the AF of LFM-Costas. Moreover, the AF sidelobe levels of GSFM pulse train were high. Due to these disadvantages, we constructed an optimization model composed of three fitness functions about AF and correlation properties. The characteristic parameters of pulse trains were optimized by means of a GA algorithm. Optimization results showed that the Doppler ambiguity was reduced drastically in the optimized LFM-Costas. Moreover, the optimized GSFM not only retained the ideal thumbtack AF mainlobe, but also reduced AF sidelobe levels. Better correlation properties were also achieved via the optimization model. In addition, the optimized pulse trains exhibited improved performance on reverberation suppression.

We have identified three potential avenues for future works. The first is to utilize the optimization approach on other kinds of active sonar waveforms, such as some phase-modulated waveforms worth being optimized. The second is to expand the analytical method and optimization approach to wideband transmitted waveforms, which are more suitable for engineering application. The third is to verify the performance of our optimization model through sea-trial experiments.

## Figures and Tables

**Table 1:** The sidelobe levels of LFM-Costas pulse train (dB).

Objects	Average AF Sidelobe	AF Sidelobe Peak	Average ACF Sidelobe	ACF Sidelobe Peak
Initial	−48.89	−1.83	−87.29	−36.44
Optimized	−49.02	−18.99	−99.37	−42.43
